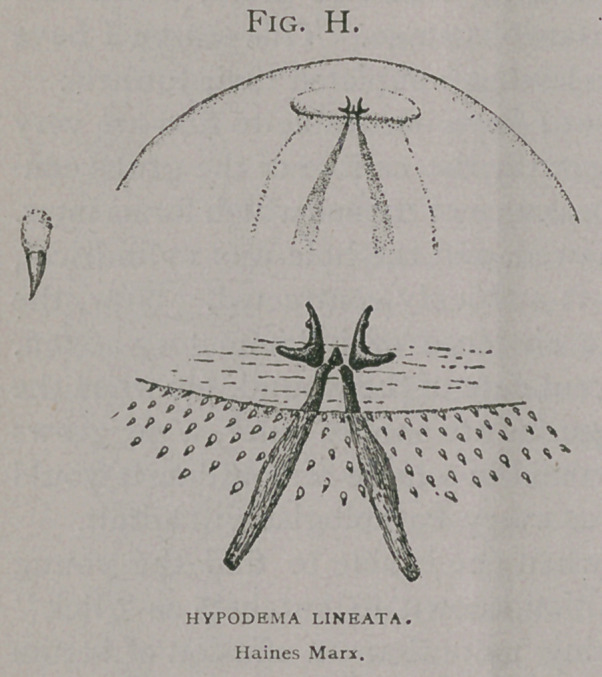# The Oxwarble of the United States

**Published:** 1891-06

**Authors:** Cooper Curtice

**Affiliations:** Veterinarian; Moravia, N. Y.


THE JOURNAL

OF

COMPARATIVE MEDICINE AND

VETERINARY ARCHIVES.

VOL. XII. JUNE, 1891. No. 6.

### THE OXWARBLE OF THE UNITED STATES.

BY COOPER CURTICE, VETERINARIAN.
Moravia, N. Y.

So much has been written, and I may add re-written, concerning the Oxwarble that it would seem that we should now know all about these wonderful pests. I must confess that my astonishment was as great as anyone's when I found, it is now three years since, that comparatively little was known about this, the commonest cattle parasite.

On March 14, 1891, I placed a number of grubs taken from the hides of slaughtered cattle, under favorable conditions for metamorphosing. Within two days some of the darkest colored assumed that peculiar form which indicated that histolysis had begun. About noon April 16, two flies, a male and female, had emerged from two of the cases.

An analytic comparison of these flies with Brauer's (1) description determined these to be *Hypoderma lineata* Villers. Through the kindness of the Curator of Entomology of the U. S. National Museum, Prof. C. V. Riley, I have, with his able assistant, Mr. Th. Pergandi, compared these bred specimens with the fourteen specimens in that collection, and especially with the specimen identified by himself (5), and the distinguished dipterologist, Mr. S. W. Williston. They proved to be of the one species, *H. lineata*. There were slight differences between the sexes in color, length of hair, breadth of forehead, size of abdomen, etc., but these were sexual.

As *Hypoderma lineata*, Villers, is but little known in this country, a review of the little literature at hand concerning its occurrence in the United States will prove of interest.

1863. Brauer (1) records *H. lineata* from Kentucky, from an adult in the Imperial Museum, Vienna.

1875. Brauer (2) describes and figures a larva *H. Bonassi*, taken from the back of a buffalo in Colorado.

1886. An observer (3) records the grub of 'heel-fly' as a native of Texas, and as having certain differences from *H. bovis*, and details life history.

1886. Williston (4) records *H. lineata* from Northern California and Arizona from specimens in his collection.

1887-88. S. O. Cotton (5) records a connection between the heel-fly and the grubs of cattle-backs.

1889. Riley (6) identified a specimen sent to him by W. F. M. Dickson, Milford, Texas, as *H. linearis*, Villers, and connects this species with the 'heel-fly' of Texas.

1890. Brauer (7) describes specimens of grubs taken from cattle in Europe as larvæ of *H. lineata*, for the reason that adult *H. lineata* were taken in the vicinity and no other larval form was known. He also connects these larvæ with *H. Bonassi* and cites the occurrence of adult *H. lineata* in America.

1891. Holstein* (11) records, April 25th, rearing the Texas Heel-fly.

1891. Curtice (12) records May 9th, the epitomizes life-history of the Heel-fly. The writer (present article) rears two specimens, a male and female, of *H. lineata* from the cattle grubs taken in Washington, D. C.

The expected outcome of *Hypoderma lineata* from the cattle grubs has been indicated to me by the following facts:

1. No adults of *Hypoderma bovis* have ever been recorded as captured in this country, all have been *H. lineata*.

---

* To the untiring enthusiasm and scientific tastes of Mr. George Wolf Holstein, a well-known ranchman of Albany, Texas, I am indebted for a specimen of *Hypoderma lineata*, hatched April 15, 1891, which he has reared from the larval stage, secured from an animal on his ranch. He states that the grub metamorphosed in about twenty days, and having shown it to numerous fellow-ranchmen, also adds, that they agree with him that the fly is the well-known and dreaded heel-fly. While I do not now propose to give my line of reasoning, I take pleasure in stating that all facts that have come to me demonstrate this most completely.

2. No original figures of *H. bovis* drawn in this country, have yet appeared.

3. All American writings concerning the cattle-grubs have evidently been drawn from European sources together with figures; and few actual observations of biology or of detailed anatomy of adult or larva have been made.

4. Of five hundred specimens of matured larvæ in the Bureau collection, from the North Eastern United States, all vary but little among one another, resemble the larva described by Brauer (2) as *H. Bonassi*, and present specific differences from the descriptions of the larva of *H. bovis*. For the last two years my search has been for larvæ of *H. bovis*, which is said to be so plentiful throughout the country.

Besides other characters the principal one separating the larva of *H. bovis* from *H. lineata*, is the complete absence of spines from the tenth segment and from the ninth, excepting the ventral area in the former, while in the latter these segments carry spines, especially on their caudal edges.

Diagrams showing the exact relations of Brauer's (6) *H. bovis* (Fig. *a*) and *H. lineata* (Fig. *b*) to the American species, (Fig. *c*), are introduced, for the use of future observers. These diagrams really present the characters better than any description, or any other kind of figures. They are due to the ingenuity of Prof Brauer, and are so complete that I gladly adopt them, as I am sure others will in turn whenever describing this class of insects.

Fig. A.

HYPODERMA LINEATA VILLERS.

After Brauer.

In the first column are the spines of the dorsal area; in the second, of the dorso-lateral; in the third, the lateral; in the fourth the ventro-lateral and in the fifth, of the ventral. Each column is further sub-divided into twelve horizontal divisions, each representing a segment of the larva, and the first two, the cephalic, being counted as one. The spines are indicated by dots placed in the cephalic and caudal portion of these rings as they occur on the grub.

In comparing these diagrams it will be seen that the variations between that of *H. Bonassi* (Fig. *i*), and the American *H. lineata*, (Fig. *k*) and the European *H. lineata* are less than between either and *H. bovis* (Fig. *l*).

FIG. B.

HYPODERMA BOVIS.

After Brauer.

About a year ago I published (7) an account of the life-history of the larvæ *Hypoderma bovis.* I must confess that I was not then dealing with *H. bovis.* I had not at the time complete data for establishing the new species and preferred to present the matter under the accepted name for the species.

I regard the life-history of *Hypoderma lineata* to be as follows: The adult fly lays its eggs somewhere on cattle presumably the back, by attaching them to the hairs. This attachment is admirably outlined by the structure of the egg, (Fig. *d*) which is similar to that of the *horse bot-fly Gastrophilus equi*, and by the structure of the ovipositor, which is not adapted for boring. While some authors have contended that the egg is laid in the skin others have conclusively shown that this is not the case.

FIG. C.

HYPODERMA LINEATA VILLERS.

Haines, original.

Development takes place within the egg while yet attached to the hair, as demonstrated by the late Dr. Handlirsch

Fig. K.

Fig. I.

HYPODERNA BONASSI.
After Brauer.

HYPODERNA LINEATA.

Haines, original.

Fig. L.

HYPODERNA BOVIS.
After Brauer.

(7) whose figure of the larva within the egg case (Fig. *e*), Dr. Brauer so clearly described.

Fig. D.

EGG OF HYPODERMA LINEATA.

Haines.

From this point on my version of the life-history varies from that of others until the larva has arrived at its destination in the cysts, under the skin which open to the air through the hide. As it becomes necessary to designate the different stages through which the larva passes, names for our present use must be adopted. The first stage as shown by Brauer (7) is undoubtedly the form that emerges from the egg-shell—the oval-larva. The next known stage is that found in the œsophagus, the œsophageal (Fig. *f*), whether this stage may be shown to be different from the oval or whether intermediate stages may be found is yet to be proven. The œsophageal stage is identical with the subcutaneous and with the first form found in the skin tumors. So as not to confuse the three stages found in the skin by giving them different names from those used in my earlier article (7) we may call the three stages the first, second and third cutaneous stages.

Fig. E.

LARVAL HYPODERNA LINEATA IN EGG SHELL.

After Handlirsch.

It has been stated by various authorities that the young grub emerging from the shell bored its way through the skin until it reached the subcutaneous tissue, and thus made its channel. From circumstantial evidence I believe that the embryos are licked by the cattle and swallowed, or lodged in the back of the mouth or œsophagus. This theory is based on the appearance of the cattle grubs in the walls of the œsophagus in November, long before they are found in the backs of cattle in this locality. Later, about Christmas time, the grubs appear suddenly, and in full force under the skin of the back.

At their first appearance under the skin (Fig. *f*). they are as large as those found in the œsophagus at that time and differ in no wise from them. By the latter part of January or early in February all have disappeared from the œsophagus together with all traces of inflammatory action in that organ so observable in January.

Fig. F.

HYPODERMA LINEATA.

Haines.

He stated in the above cited article (7) larvæ had been found next the eleventh rib on the thoracic side; also by Huirichsen (9) in the spinal canal; in subcutaneous muscles, Brauer (1), and in subcutaneous connective tissues by myself (8). I have, in addition, found Nov. 7, 1890, a specimen in the connective tissues immediately adjacent the spleen. Twice have wounds in the œsophageal muscular coats occurred to me, which I believe to have been caused by the larvæ while penetrating it. A year ago I discovered some small spots which seemed to me to have been gnawed on the underside of freshly removed hides, which also carried the larva in the first cutaneous stage. This season I have not found any, all of the grubs having completed their tunnels.

The earliest grub holes that I have been able to find are very uniform in size corresponding with the calibre of the grubs contained in them, and had no appearance of the sac which forms later. The walls were rough as if gnawed, and the hole was cylindrical, to near the epidermis, when it suddenly contracted. Now, the freshness of the wound and the abscence of inflammatory action, is a very good index of the recent date of the wound, for when the wound is exposed to the air germs are sure to enter, a sac grows and secretes pus. Were the wound of a more remote date it would be of quite another character, as every Pathologist will admit.

Just preceeding the time when one is able to find the young warbles in the skin, that condition known to butchers as "lick" appears. The "lick" is nothing more than an effusion of serum into the connective tissue membrane, and is produced by the inflammation set up by the wanderings of the young grubs. This effusion can also be found in the walls of the œsophagus, just prior to the final disappearance of the grubs. The disappearance of the "licks" from the tissues underlying that portion of the hide most infested, the saddle, is followed by finding the grubs in sacs in the first and second cutaneous stages. When the sacs are well formed the "licks" have disappeared.

These "licks" are said by farmers and butchers to be caused by cattle licking themselves. It is, easy to understand, however, that the cattle lick themselves at this time on account of the irritation produced by the grubs in piercing through the sensitive skin. The appearances of "lick" in those parts where the force of the tongue could not reach, as in the œsophagus, an appearance which has been my guide to the grub and its vicinity, is quite good proof that the grubs cause "lick."

FIG. G.

HYPODERMA LINEATA.

Marx.

The grubs bore through the skin caudal end (Fig. *g*), first. This end is best provided with the proper apparatus for tearing the skin fibres—numerous rows of short, stout spines. These, Fig. *e*, Brauer (1) has figured and Ormerod (10); Brauer (7) figures spines on the ova-larva, by which the young larva enters the œsophagus, caudal end first. It is true that there are a few spines and two hooks on the head (Fig. *h*), in the earlier stages but these are insignificant as compared with those of the caudal end.

FIG. H.

HYPODEMA LINEATA.

Haines Marx.

Having made its tunnel through the hide the larva moults. The differences between the first and second cutaneous stages also adds another proof to strengthen the net of circumstantial evidence pointing out this life-history. The breathing pores of the first cutaneous stage are quite small and no larger than in the œsophageal stage or subcutaneous. The stigmatal plates of the second stage, however, are much larger and the respiratory tract quite plain. This indicates that while there may have been limited respiration in the early stages, that so soon as the grub reaches to the air a larger respiratory apparatus is not only necessary, but it acquires it.

The difference in the food between the subcutaneous stage and the first cutaneous stage, or certainly the second stage, is quite marked. Before the tunnel is made or completed the contents of the alimentary canal are yellow, like the inflammatory effusion it excited, but after the sac forms, after it looses the mouth hooks in the second stage, the contents become much darker like the pus secreted from the lining membrane of the sac.

The different stages of the larvæ in the skin cysts can be easily connected by dissecting out the whole cysts, marking their contents, and finding the moults of the earlier stages. Miss Eleanor Ormerod, of Torrington House, St. Albans, England, Consulting Entomologist of the Royal Agricultural Society, has figured and described the three cutaneous stages of *H. bovis*, and published the most practical economic work of anyone; and I am indebted to her for a series of her valuable writings on this subject.

On completing the larval stages of its life the grub forces its way out through the narrow hole by means of vermicular contractions and the stout spines. Falling to the ground, it may force its way into some crevice or under the edge of some adjacent object, and then passes into its pupul state. The specimens I bred occupied six weeks in transforming. There were bred by Mr. Holstein about three. Metamorphosis completed, the mature fly emerges through the hole left by a cap splitting, from the side of the head end.

Fortunately, the female-fly was detected in the act of laying an egg, Fig. *d*, the attaching portion of the egg came first. The time of deposition, the exact place or places of deposition, and the discovery of larva less than 7 mm. in length have yet to be investigated.

The results of this paper were obtained while prosecuting investigations upon the animal parasites of cattle, while I was in the employ of the Bureau of Animal Industry, Department of Agriculture. If of value, they show the importance of employing specialists to work up the life-histories of cattle parasites, and continuing their work through a series of years, or until it becomes fairly well demonstrated that they cannot be pursued further profitably.

The material is in the Bureau collection. It consists of over 200 œsophageal grubs; 45 of the first, 150 of the second and 550 of the third cutaneous stages. So far but three flies have been bred. I am indebted to Dr. D. E. Salmon, the Chief of the Bureau, for the use of material, and for drawings of which I have had copies made.

### LITERATURE.

1. Brauer, Monographie d. Oestriden, 1863.
2. Brauer, Besch. neuer und ungenügend bekannter Phryganiden und Oestriden Verh'dl d. k. k. Zool. Bot. Ges. Wein Bd xxv, p. 75 Taf. iv, fig. 2 and 2a, 1875.
3. Correspondent in Kansas City Live Stock Record, May 20, 1886. Abstract from Pueblo Live Stock Review and Standard.
4. Williston, Trans. Amer. Ent. Soc., Philadelphia, vol. xiii, p. 307, 1886.
5. S. O. Cotton, Fourth and Fifth Rept. Bureau of Animal Industry, U. S Dept. Agri, p. 493, 1887-88.

6. C. V. Riley, Insect Life, U. S. Dept. Agriculture, vol. i, p. 318, 1887.
7. Brauer u. Handlirsch, Ueber d. Festellung des Wohnthieresden *Hypoderma lineata* Villers, etc. Verhdl d. k.k. Zool. Bot. Ges. Wien, Oct. 1890, Bd. xl, p. 509, 3 fig.
8. Curtice, Insect Life, U, S. Dept. Agriculture, vol. ii, No. 7 and 8, 1890. Larvæ of *Hypoderma bovis ?*
9. Hurichsen, Archiv f. wissen prak Thierheilkunde Bd. xiv, p. 219, 1888. Ueber eines neuen Parasiten im Ruckenmarkskanal des Rindes.
10. Ormerod, Warbles of Cattle, 11th. Ann. Rept. p. 116, and other reports.
11. Holstein, Texas Live Stock Journal, April 25th, 1891.
12. Curtice, *Ibid*, May 9th, 1891, p. 14.

## Figures and Tables

**Fig. A. f1:**
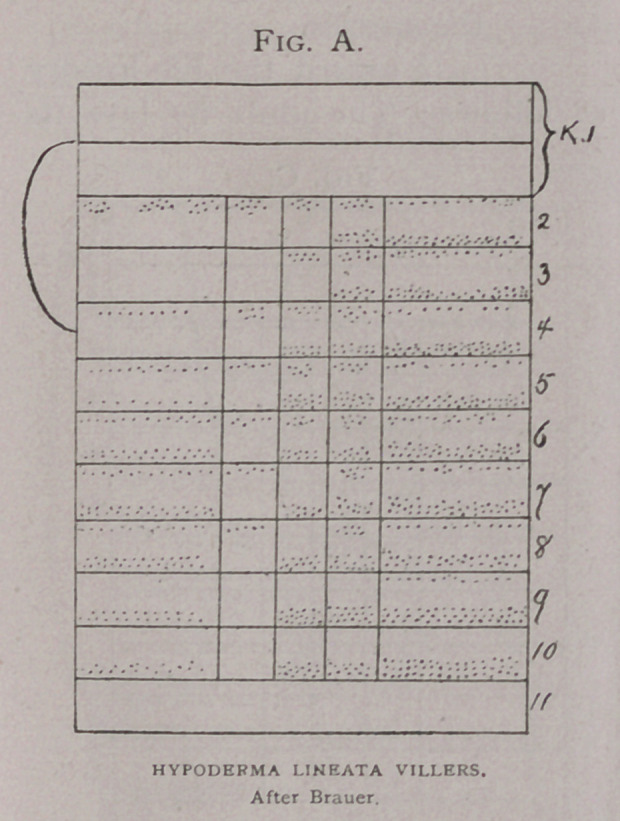


**Fig. B. f2:**
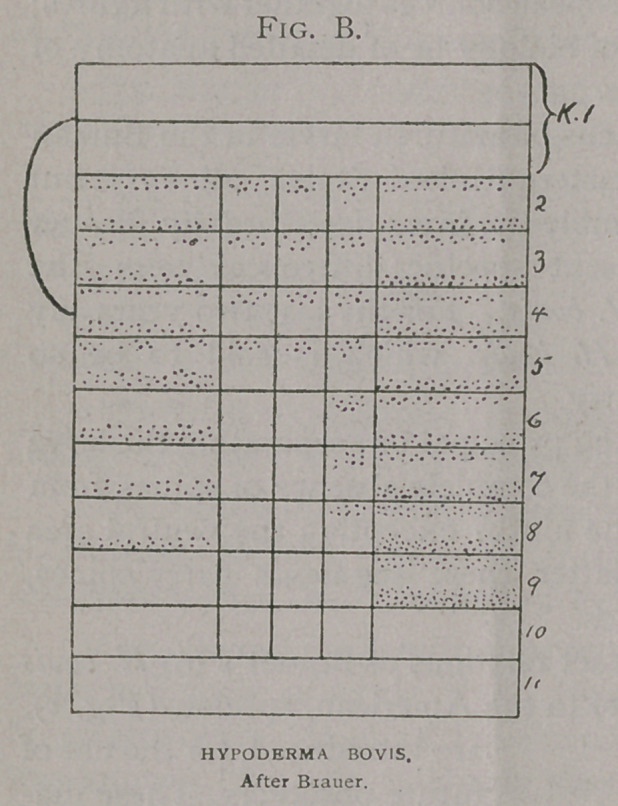


**Fig. C. f3:**
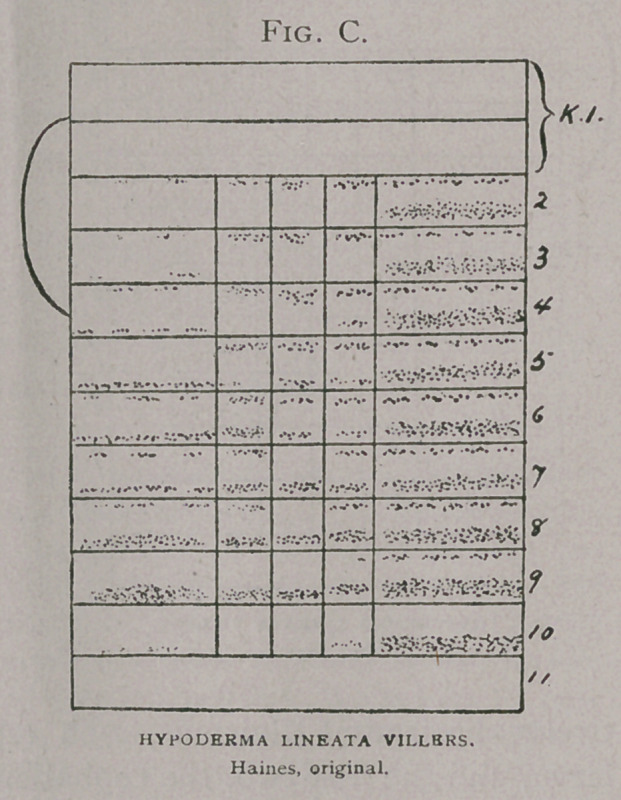


**Fig. I. f4:**
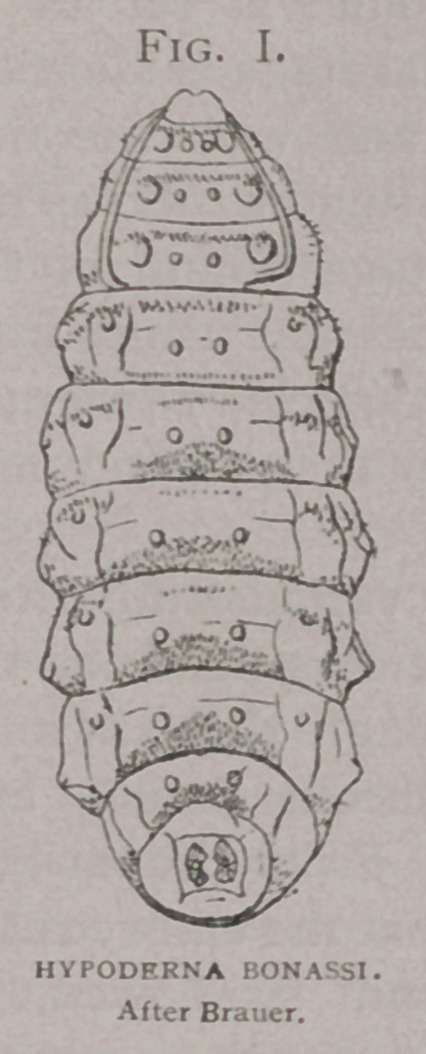


**Fig. K. f5:**
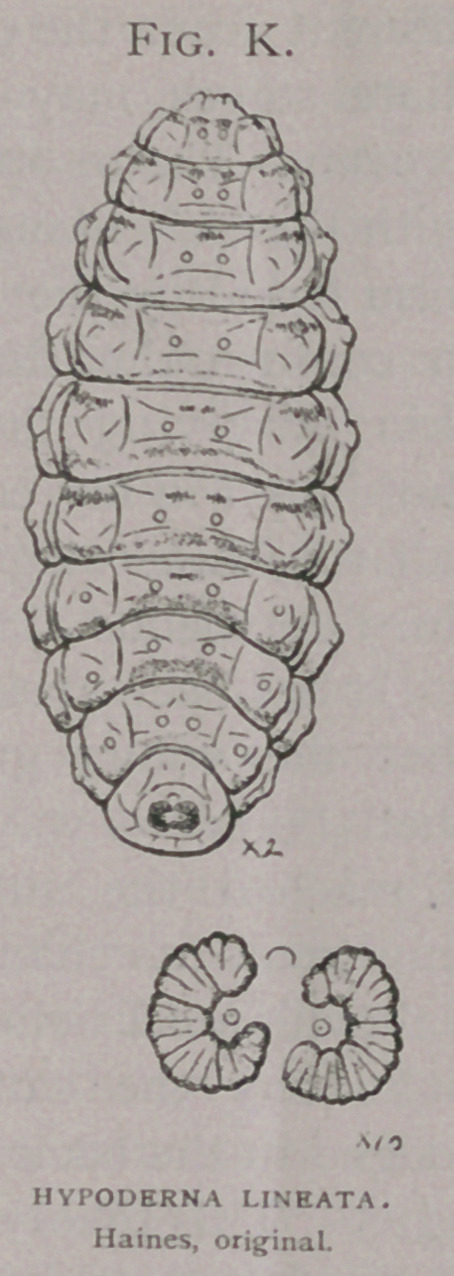


**Fig. L. f6:**
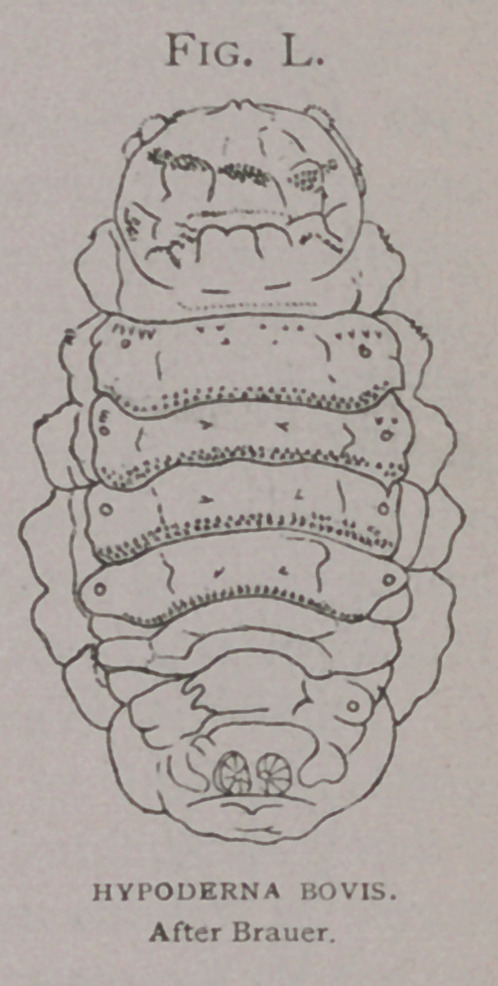


**Fig. D. f7:**
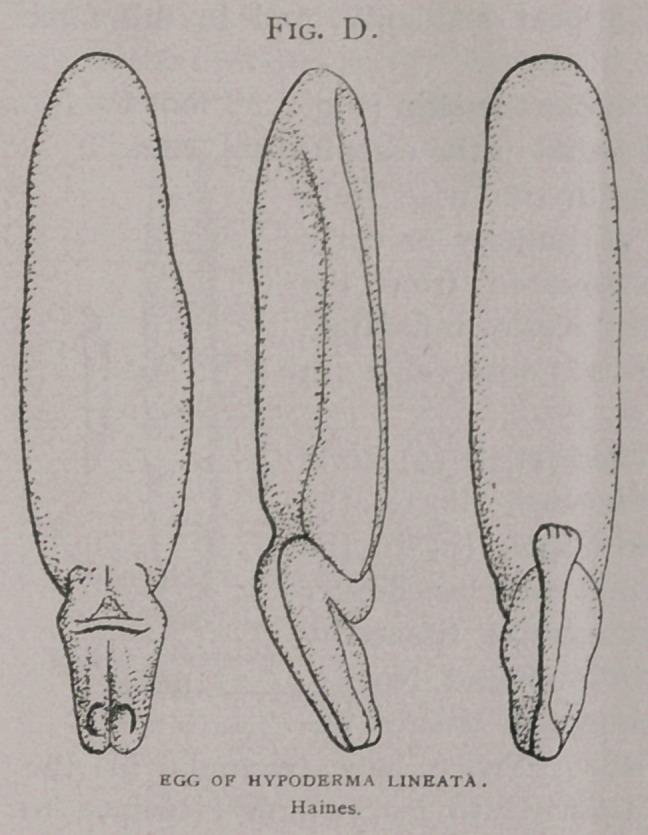


**Fig. E. f8:**
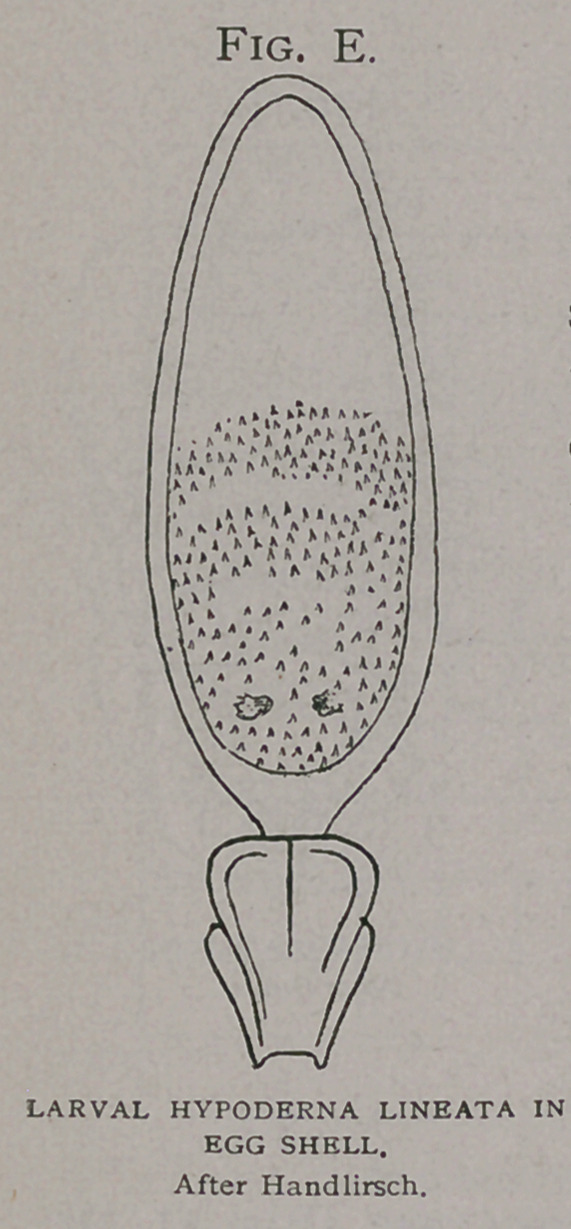


**Fig. F. f9:**
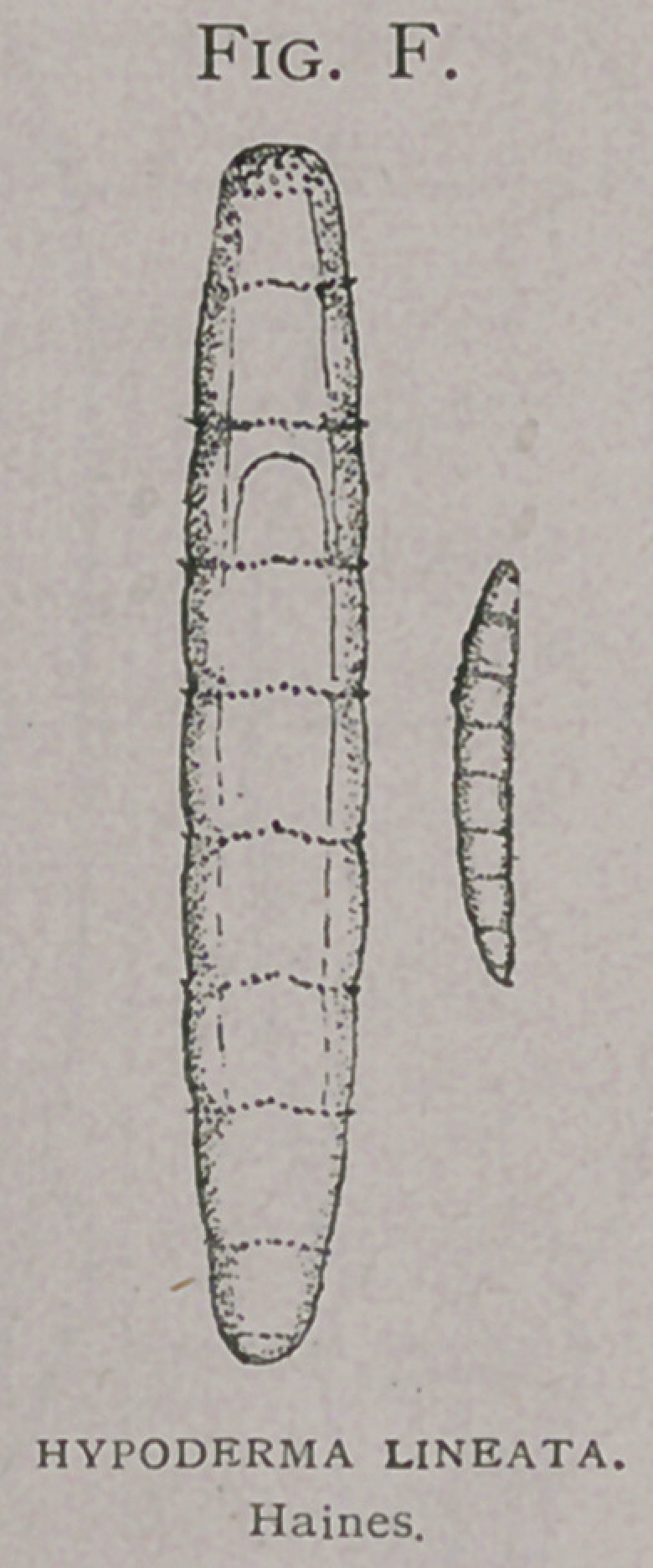


**Fig. G. f10:**
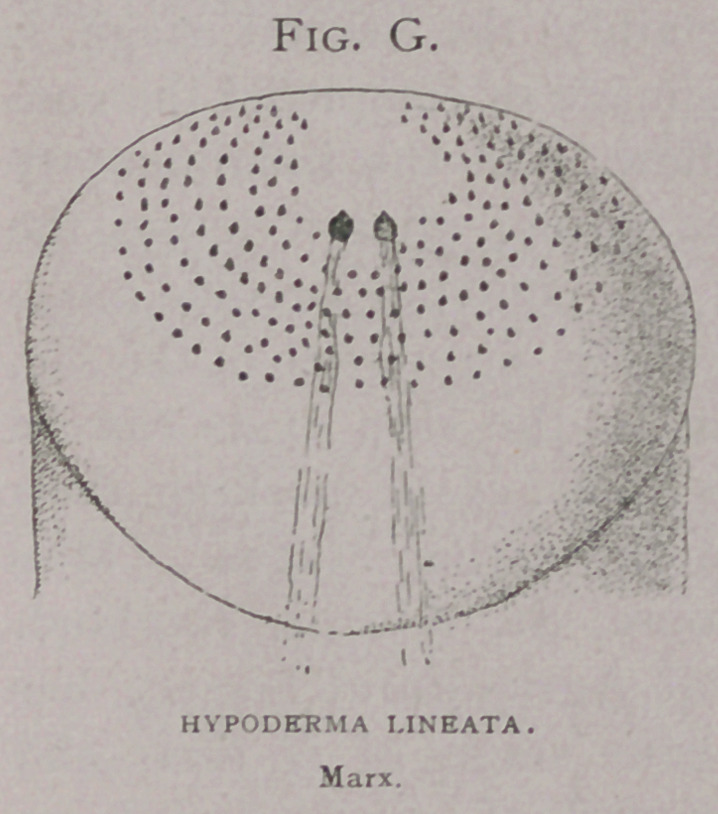


**Fig. H. f11:**